# Comparison of clinical outcomes in critical patients undergoing different mechanical ventilation modes: a systematic review and network meta-analysis

**DOI:** 10.3389/fmed.2023.1159567

**Published:** 2023-08-22

**Authors:** Mengyu Wu, Xiaohong Zhang, Yu Jiang, Yun Guo, Wenjing Zhang, Hong He, Yanhua Yin

**Affiliations:** ^1^Department of Critical Care Medicine, Xiangyang Central Hospital, Affiliated Hospital of Hubei University of Arts and Science, Xiangyang, Hubei, China; ^2^Nursing Department, Xiangyang Central Hospital, Affiliated Hospital of Hubei University of Arts and Science, Xiangyang, Hubei, China; ^3^Department of Nursing, School of Nursing, Wuhan University, Wuhan, Hubei, China

**Keywords:** critical illness, mechanical ventilation, network meta-analysis, systematic review, mortality

## Abstract

**Purpose:**

To evaluate the effects of different mechanical ventilation modes on critical patients.

**Methods:**

PubMed, Embase, Web of science, and Cochrane Library databases were searched from their inception to November 15, 2022 for randomized controlled trials on the application of different mechanical ventilation modes in critical patients. Two researchers independently screened the literature, extracted data, and assessed the risk of bias in the included studies. R4.2.1 was used for this network meta-analysis.

**Results:**

Twenty-eight RCTs involving 3,189 patients were included. The interventions in these RCTs included NAVA (neurally adjusted ventilatory assist), PAV (proportional assist ventilation), ASV (adaptive support ventilation), Smartcare/PS (Smartcare/pressure support), PSV (pressure support ventilation), PSV_ATC (pressure support ventilation_automatic tube compensation), and SIMV (synchronized intermittent mandatory ventilation). The network meta-analysis showed that, compared with the PSV group, there was no significant difference in duration of mechanical ventilation, duration of ICU stay, and hospital stay between NAVA, SIMV, AVS, PAV, Smartcare/PS, and PSV_ATC groups. Compared with PSV, PAV improved the success rate of withdrawal of ventilator [OR = 3.07, 95%CI (1.21, 8.52)]. Compared with PSV and PAV, NAVA reduced mortality in the ICU [OR = 0.63, 95%CI (0.43, 0.93); OR = 0.45, 95%CI (0.21, 0.97)].

**Conclusion:**

NAVA can reduce mortality in ICU, and PAV may increase the risk of withdrawal of the ventilator. There was no significant difference between PSV and other mechanical ventilation modes (NAVA, SIMV, AVS, PAV, Smartcare/PS, and PSV_ATC) in the duration of mechanical ventilation, duration of ICU stay, or hospital stay. Due to the limitations, more high-quality studies are needed to verify these findings.

## 1. Introduction

Critical patients are characterized by acute onset, rapid change, and a high mortality rate, mainly manifested as acute hypoxic respiratory insufficiency or respiratory failure. General treatment often fails in treating this condition and mechanical ventilation is the main approach (1). As more intensive care units (ICUs) have been built in recent years, ventilators have been widely applied, and mechanical ventilation has showed remarkable effects in the treatment of critical patients (2). Mechanical ventilation is an approach to airway maintenance that uses a ventilator. Generally, mechanical ventilation utilizes mechanical devices to change, control, or replace the patients’ spontaneous breathing movement. Mechanical ventilation can improve ventilation and oxygenation, prevent the accumulation of carbon dioxide, and ameliorate hypoxia, thus preparing critical patients for further treatment (3, 4). The mechanisms of different mechanical ventilation modes and their effects on patients varied greatly. In current clinical practice, ventilation modes for critical patients include NAVA, SIMV, PSV, AVS, PAV, Smartcare/PS, and PSV_ATC (5, 6). Although mechanical ventilation has assured benefits, long-term invasive mechanical ventilation could probably induce lung infection, lung volutrauma, atelectrauma, tracheal injury, biological damage, barotrauma, oxygen poisoning, and diaphragmatic insufficiency (7). Moreover, it is challenging to stop using the ventilator in time. Extending the time to withdrawal of mechanical ventilation can increase the mortality and hospitalization time, result in high treatment costs, and increase the economic burden of patients. Therefore, it is of an urgent need to find the most effective mechanical ventilation mode in clinical practice (8).

At present, there is no definite agreement on which mode to use for mechanical ventilation in critical patients (9). This study aimed to resolve the disputes via network meta-analysis to provide potent evidence for the clinical decision of choosing the most effective treatment.

## 2. Materials and methods

### 2.1. Literature retrieval

PubMed, Embase, Cochrane Library, and Web of science databases were searched for RCTs on the use of different mechanical ventilation modes for critical patients. The retrieval was as of November 15, 2022. Subject terms and free words were used, and the search terms included (Interactive Ventilatory Support OR Proportional Assist Ventilation OR Assist Ventilation, Proportional OR adaptive support ventilation OR Smartcare/PS) AND (Critical Illness OR Intensive Care Units). The specific search strategies are shown in Supplementary Table S1.

### 2.2. Inclusion and exclusion criteria

Inclusion criteria: critical patients (>18 years of age) admitted to the ICU due to a critical illness; treatment with mechanical ventilation (including NAVA, SIMV, PSV, AVS, PAV, Smartcare/PS, PSV_ATC); the primary outcome measures were duration of mechanical ventilation, duration of ICU stay, hospital stay, and ICU mortality; and the secondary outcome measure was withdrawal of the ventilator.

Exclusion criteria: conference abstract, protocol, letter, duplication, system review, full text unavailable, data unavailable, and animal experiments.

### 2.3. Data extraction

Two independent reviewers screened the literature to extract data. The titles and abstracts of the literature were reviewed. Professionals of the related area were consulted if the reviewers had disagreements on the articles. The full texts of the articles were downloaded for screening. The inclusion and exclusion criteria were strictly followed in the literature screening. The outcome measures of the two groups were extracted and cross-checked to ensure the consistency of the extracted data. The extracted data mainly included the first author’s name, year of publication, country, sample size, gender, age, patient characteristics, and outcome measures.

### 2.4. Quality evaluation

The quality evaluation of the included studies was completed independently by 2 researchers. The quality of the included studies was evaluated using the bias evaluation tool of Cochrane Handbook for Systemic Reviews of Interactions 5.1.0. The evaluation included 7 items: random sequence generation (selective bias), allocation concealment (selective bias), blinding to the implementer and participant (implementation bias), blinding to the outcome evaluator (observation bias), result integrity (follow-up bias), selective reporting of study results (reporting bias), and other sources of bias. Each included study was evaluated according to the items above. The studies were rated as “low risk” if all the criteria above were met, indicating that the overall risk of bias was low and the study quality was high. The studies were rated as “unclear risk” if part of the criteria above were met, indicating that there was a moderate risk of bias. The studies were rated as “high risk” if none of the criteria above were met, indicating that there was a high risk of bias and the study quality was low.

### 2.5. Statistical analysis

The gemtc software package in R 4.2.1 software and the JAGS software were used to conduct this network meta-data analysis and plot a network evidence map as well as a probability ranking map. The point and interval estimations were used as indicators for effect size. The continuous variables (hospitalization duration, ICU duration, and mechanical ventilation duration) were presented as standard mean difference (SMD) with 95% confidence interval (CI). SMD < 0 indicated that one ventilation mode might be inferior to the other, and SMD > 0 indicated that one ventilation mode might be more effective than the other. The inclusion of 0 in the 95%CI indicated no significant difference. The discrete variables (ICU mortality and withdrawal of the ventilator) was presented as odds ratio (OR) with 95% CI. OR < 1 indicated that one ventilation mode might be inferior to the other, and OR > 1 that one ventilation mode might be more effective than the other. The inclusion of 1 in the 95% CI indicated no significant difference. In this study, Bayesian Markov–Monte Carlo random-effects model was used to quantitatively synthesize the results. Five chains were used for simulation, with 5,000 pre-iterations and 20,000 iterations. When there was a closed loop in the evidence network, the node splitting method was used for the inconsistency test to verify whether the direct comparison was consistent with the indirect comparison. *p* > 0.05 indicated that there was no significant inconsistency between the direct and indirect comparisons; otherwise, there was statistically significant inconsistency. The probability ranking map was drawn and the priorities of different interventions were ranked according to the SUCRA values.

## 3. Results

### 3.1. Literature screening and results

**Figure 1:**
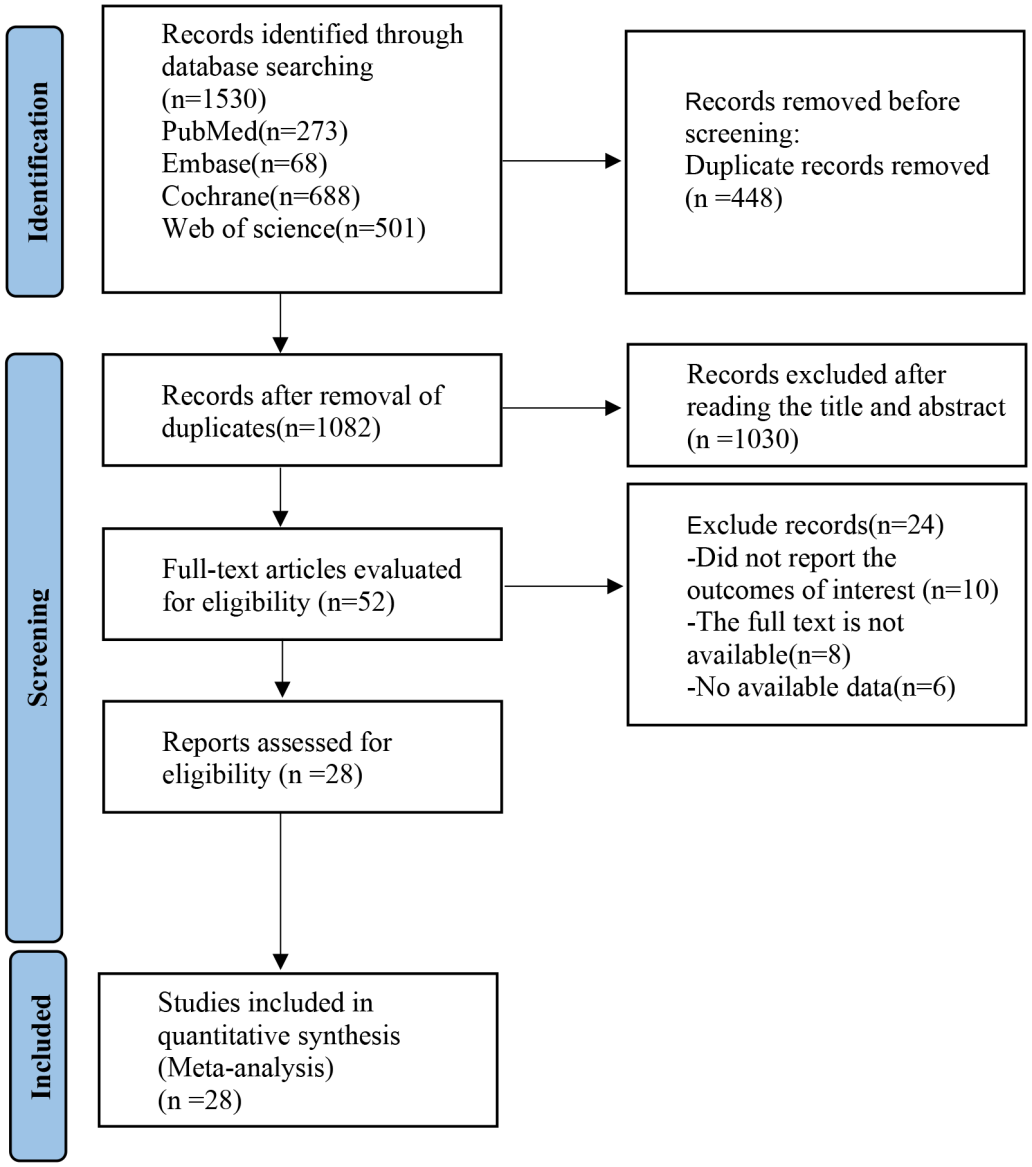
Basic feature table of articles.

PubMed, Embase, Cochrane Library, and Web of Science databases were searched and a total of 1,530 articles were initially retrieved. 1,082 articles were obtained after removing the duplication. Fifty-two articles were excluded by reading the titles and abstracts, and 28 articles were finally obtained by reading the full text. The detailed screening process is shown in Figure 1.

### 3.2. The table for the basic characteristics of the included articles

A total of 28 (10–37) RCTs were included, involving 3,189 patients and interventions such as NAVA, SIMV, PSV, AVS, PAV, Smartcare/PS, and PSV_ATC. The patients were admitted to the ICU mainly due to ARDS, COPD, etc. The table for baseline characteristics is shown in Table 1.

**Table 1:** Basic characteristics of articles.

Study	Year	Country	Sample size	Gender (M/F)	Mean age (years)	Patients	Outcome
Agarwal R	2013	India	ASV:23 PSV:25	28/20	ASV:31.4 PSV:29.7	ARDS	F1; F2; F3; F4
Aggarwal AN	2009	India	PSV:18 ATC + PSV:23	29/12	PSV:26 ATC + PSV:28	Ventilatory failure due to snake bite	F1; F2; F3
Aghadavoudi O	2012	Iran	ASV:41 SIMV:40	50/31	ASV:57.9 SIMV:59.8	Undergoing elective with cardiopulmonary bypass	F1; F2
Arnal JM	2018	Switzerland	PSV:30 ASV:30	42/18	PSV:62 ASV:67	Ventilated for less than 24 h	F1; F2; F3; F6
Bernstein G	1996	United States	PSV:160 SIMV:167	193/132	PSV:6.9 h SIMV:8.1 h	Infants with respiratory distress syndrome, meconium aspiration pneumonitis	F1; F6
Bosma KJ	2016	Canada	PAV:27 PSV:23	25/25	PAV:63 PSV:67	ICU for greater than 36 h	F1; F2; F3; F6
Botha J	2018	Australia	PAV:25 PSV:24	29/20	PAV:65.1 PSV:61.15	Ventilation for at least 24 h	F1; F2; F3; F5; F6
Chittawatanarat K	2018	Thailand	SIMV:260 PSV:260	295/225	SMIV:56 PSV:56	Mechanically ventilated for at least 12 h	F2; F3
Demoule A	2016	France	PSV:66 NAVA:62	86/42	PSV:64 NAVA:62	ARDS	F1; F2; F3; F5; F6
Di Mussi R	2016	Italy	PSV:12 NAVA:13	12/13	PSV:63.2 NAVA:63.5	ARDS	F6
Elganady AA	2014	Egypt	PAV:30 PSV:30	49/11	PAV:58.13 PSV:61.2	Acute exacerbation of COPD	F1; F2; F3; F5
Fernández-Vivas M	2003	Spain	PSV:59 PAV:58	NA	PSV:65 PAV:62	ARDS	F2; F3; F6
Hadfield DJ	2020	United Kingdom	NAVA:39 PSV:38	54/23	NAVA:66.7 PSV:67.1	COPD, heart failure, ARDS	F1; F2; F3; F6
Kacmarek RM	2020	United States	NAVA:153 PSV:153	201/105	NAVA:63.9 PSV:64.7	PaO_2_/FiO_2_ < 300	F1; F6
Liu L	2020	China	PSV:52 NAVA:47	66/33	PSV:80 NAVA:75	Ventilation > 24 h	F1; F2; F3; F5; F6
Mohamed KAE	2014	Egypt	ASV:25 PSV:25	36/14	ASV:63.5 PSV:66.9	COPD	F1; F2; F5
Rose L	2008	Australia	SmartCare/PS:51 PSV:51	NA	SmartCare/PS:51 PSV:55	Ventilation > 24 h	F1; F2; F3
Sasikumar S	2013	India	PAV:13 PSV:10	16/7	PAV:51.08 PSV:45.3	Ventilated for minimum 48 h	F2
Stahl C	2009	Germany	SmartCare/PS:30 PSV:30	45/15	SmartCare/PS:66 PSV:67	Ventilation > 24 h	F1; F2; F6
Taniguchi C	2015	Brazil	SmartCare/PS:35 PSV:35	39/31	SmartCare/PS:62 PSV:66	Ventilation > 24 h	F1
Teixeira SN	2015	Brazil	PSV:46 PAV:48	58/36	PSV:44.3 PAV:41.4	Ventilation > 24 h	F2; F3; F6
Xirouchaki N	2008	Greece	PSV:100 PAV:108	138/70	PSV:63 PAV:59	Ventilation > 36 h	F5
Yazdannik A	2016	Iran	ASV:32 SIMV:32	28/36	ASV:57.9 SIMV:58.3	Ejection fraction >30% in left ventricle	F1; F3
Zhang J	2022	China	SIMV:50 ASV:50	53/47	SIMV:45.2 ASV:45.9	ARDS	F2
Dongelmans DA	2009	Netherlands	ASV:64 PSV:64	107/21	ASV:65 PSV:67	Post coronary artery bypass graft surgery,	F2
Kirakli C	2011	Turkey	ASV:49 PSV:48`	89/8	ASV:64 PSV:65	Acute exacerbation of COPD	F1; F2; F5
Kuo NY	2016	China	NAVA:14 PSV:19	24/9	NAVA:79.3 PSV:76.9	COPD patients with >21d weaning failure	F1; F3; F6
Schädler D	2012	Germany	SmartCare/PS:150 PSV:150	209/91	SmartCare/PS:67 PSV:69	Ventilation > 24 h	F3; F6

NAVA, neurally adjusted ventilatory assist; PAV, proportional assist ventilation; ATC, automatic tube compensation; ASV, adaptive support ventilation; Smartcare/PS, Smartcare/pressure support; PSV, pressure support ventilation; SIMV, synchronized intermittent mandatory ventilation; ARDS, acute respiratory distress syndrome; COPD, chronic obstructive pulmonary disease; F1, duration of mechanical ventilation; F2, duration of ICU stay; F3, hospital stay; F4, ease of ventilator use by VAS; F5, successfully weaned from the ventilator; F6, ICU mortality.

### 3.3. The evaluation of risk of bias of the included articles

**Figure 2:**
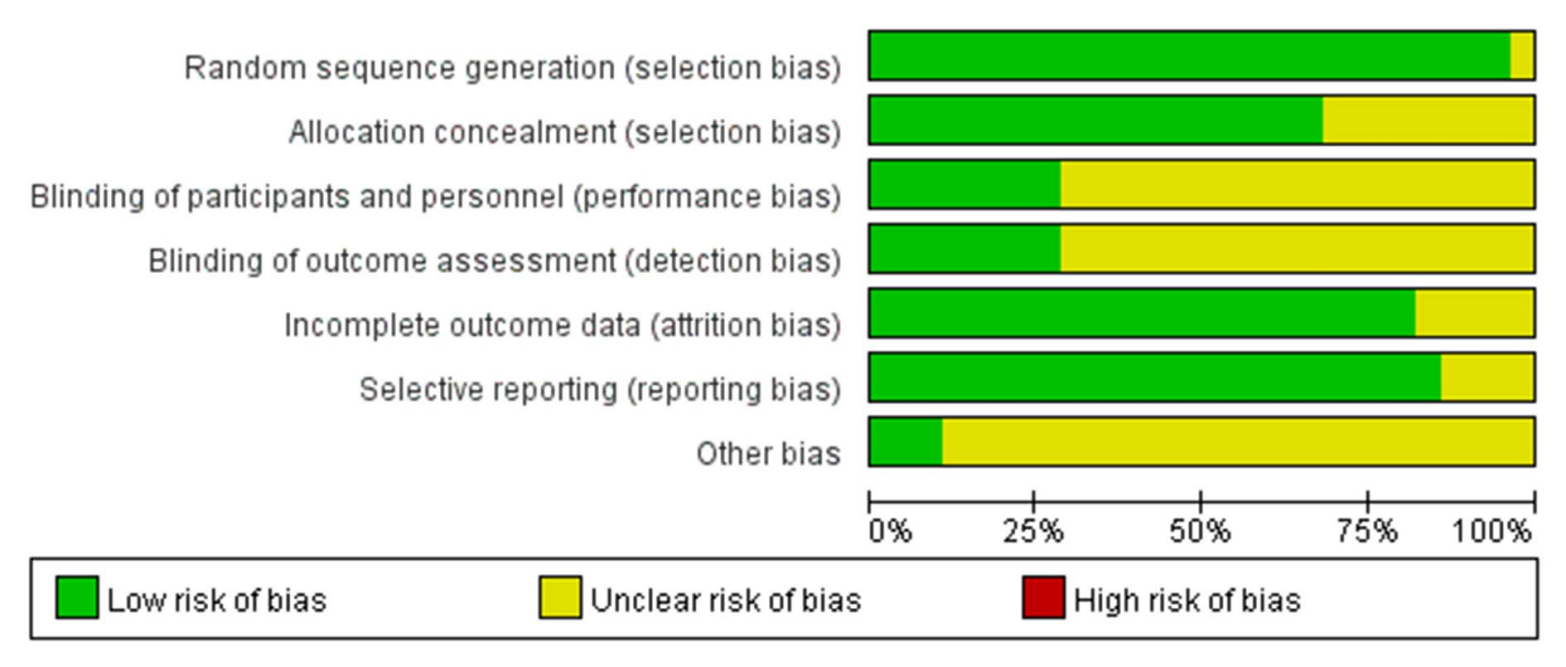
Graph for risk of bias.

**Figure 3:**
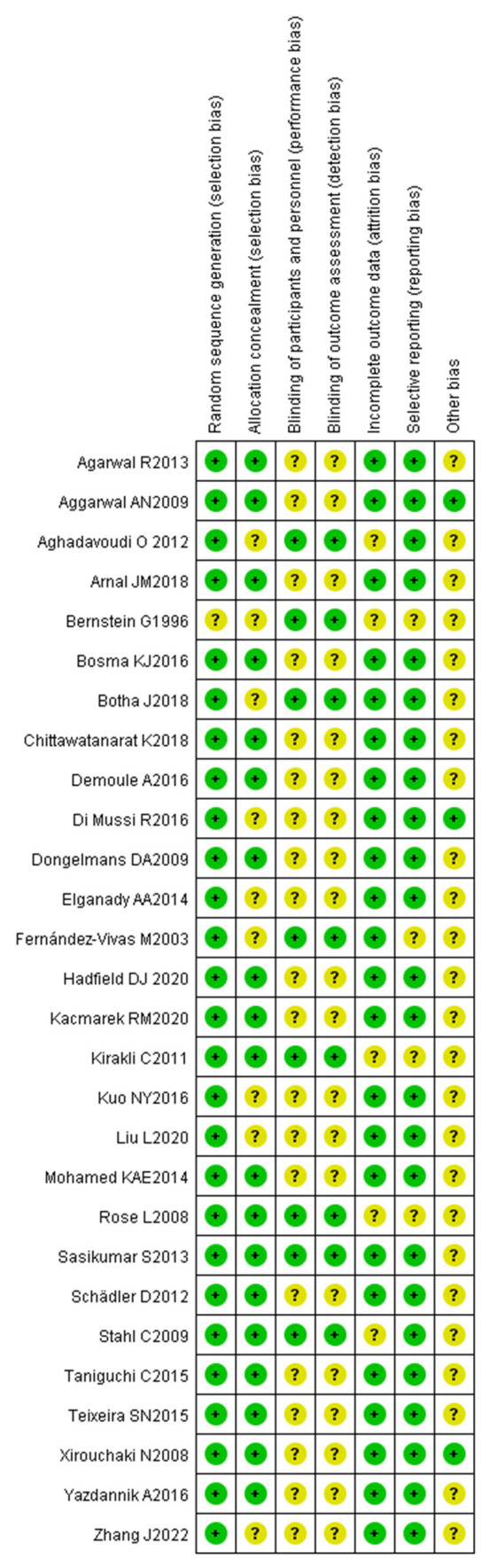
Summary for risk of bias.

The specific methods for generation of random sequences were explicitly reported in the 28 included studies, which were rated as low-risk. The main methods included a random number table and a computer-generated random number table. The basic characteristics of the included articles are shown in Figures 2, 3.

### 3.4. Network diagram

**Figure 4:**
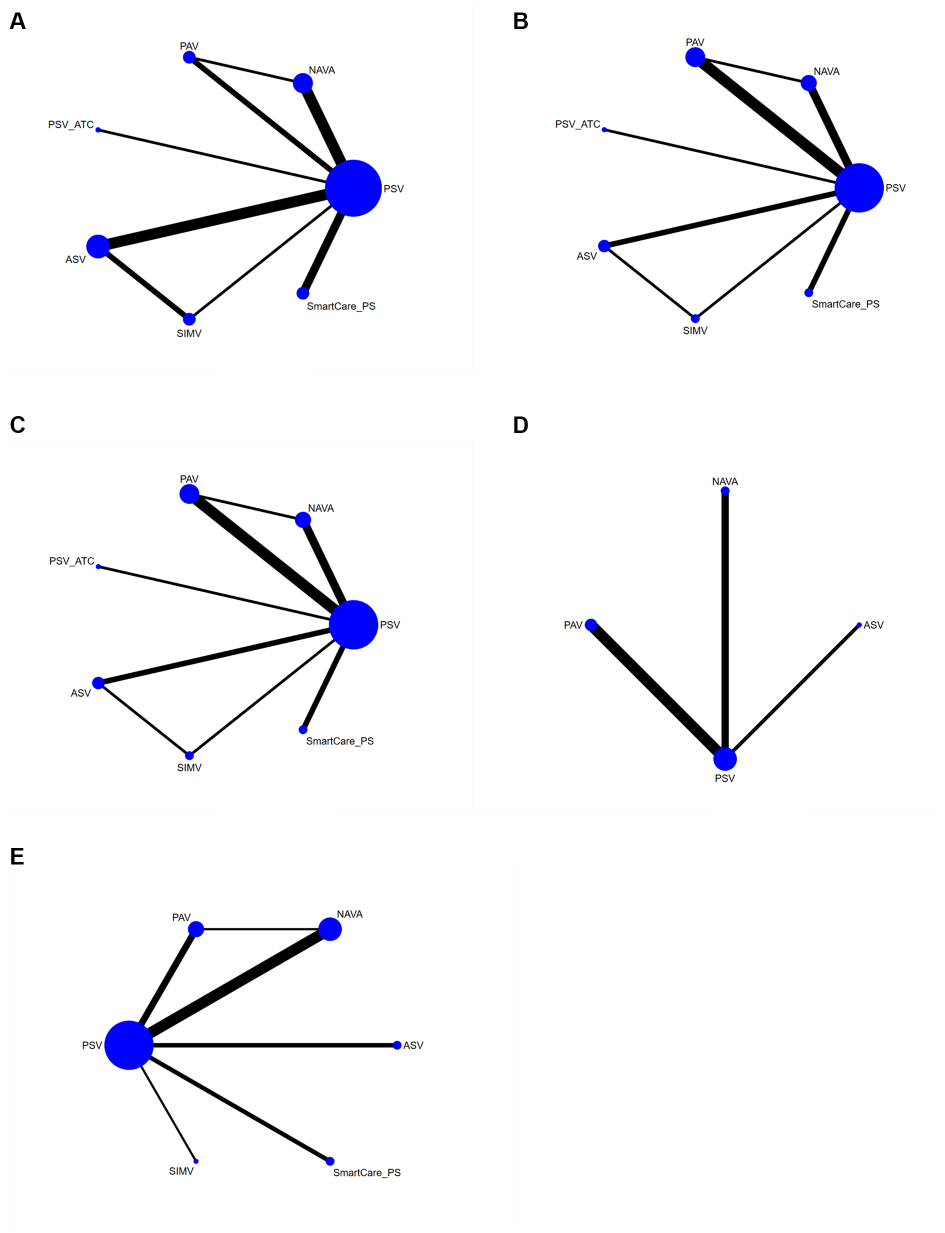
Network diagram **(A)** duration of mechanical ventilation, **(B)** duration of ICU stay, **(C)** hospital stay, **(D)** successfully weaned from the ventilator, and **(E)** ICU mortality.

The network diagram involving 5 interventions is shown in Figure 4. The spot represented the specific intervention, and the size of the spot indicated the number of patients receiving the intervention. Straight lines indicated that there was evidence of a direct comparison between the 2 interventions, and the thickness of the straight lines indicated the number of studies directly comparing the 2 interventions.

### 3.5. Network meta-analysis results

#### 3.5.1. Duration of mechanical ventilation

A total of 18 studies involving a total of 1,745 patients mentioned the duration of mechanical ventilation (the patients did not switch from invasive ventilation to non-invasive ventilation after extubating). The network meta-analysis generated 21 direct or indirect comparisons without statistical significance. This indicated that there were no significant differences in the duration of mechanical ventilation between PSV and the other interventions (NAVA, SIMV, AVS, PAV, Smartcare/PS, and PSV_ATC) (Supplementary Table S2). The interventions were ranked according to SUCRA values as follows: SIMV (83%) > ASV (79%) > NAVA (52%) > Smartcare/PS (51%) > PAV (40%) > PSV (24%) > PSV_ATC (21%) (Supplementary Table S3).

#### 3.5.2. Duration of ICU stay

The duration of ICU stay was reported in 19 articles involving a total of 1,927 patients. The network meta-analysis generated 21 direct or indirect comparisons without statistical significance. This indicated that there was no significant differences in the duration of ICU stay between PSV and the other interventions (NAVA, SIMV, AVS, PAV, Smartcare/PS, and PSV_ATC) (Supplementary Table S2). The interventions were ranked according to SUCRA values as follows: Smartcare/PS (68%) > NAVA (65%) > ASV (60%) > PSV_ATC (46%) > PAV (41%) > PSV (37%) > SIMV (33%) (Supplementary Table S3).

#### 3.5.3. Hospital stay

The hospital stay was reported in 15 studies involving a total of 1,625 patients. The network meta-analysis generated 21 direct or indirect comparisons without statistical significance. This indicated that there were no significant differences in the duration of ICU stay between PSV and the other interventions (NAVA, SIMV, AVS, PAV, Smartcare/PS, and PSV_ATC) (Supplementary Table S2). The interventions were ranked according to SUCRA values as follows: Smartcare/PS (67%) > NAVA (65%) > ASV (60%) > PAV_ATC (45%) > PAV (41%) > PSV (37%) > SIMV (33%) (Supplementary Table S3).

#### 3.5.4. Withdrawal of the ventilator

**Figure 5:**
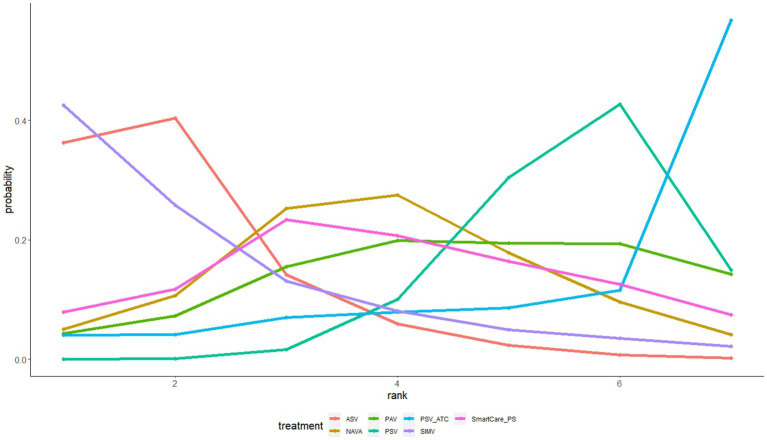
The ranking diagram of withdrawal of the ventilator.

The withdrawal of the ventilator was reported in 6 studies involving a total of 584 patients and interventions such as ASV, PAV, PSV, NAVA, etc. The network meta-analysis generated 6 direct or indirect comparisons. Compared with PSV, PAV increased the rate of withdrawal of the ventilator [OR = 3.07, 95%CI (1.21, 8.52)] (Supplementary Table S2). The interventions were ranked according to SUCRA values as follows: PAV (89%) > NAVA (58%) > ASV (40%) > PSV (13%) (Figure 5).

#### 3.5.5. ICU mortality

**Figure 6:**
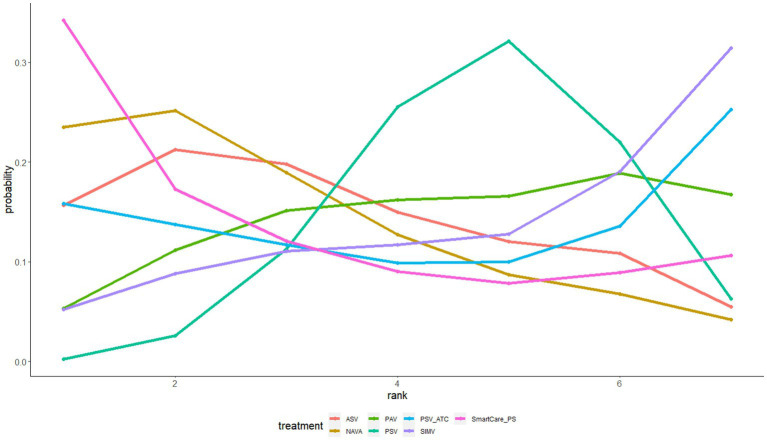
The ranking diagram of ICU mortality.

ICU mortality was reported in 14 articles involving 1,587 subjects and interventions such as NAVA, SIMV, PSV, AVS, PAV, and Smartcare/PS. A meta meta-analysis generated 15 direct and indirect comparisons. Compared with PSV and PAV, NAVA reduced ICU mortality (0.63, 95%CI (0.43, 0.93); 0.45, 95%CI (0.21, 0.97)). The interventions were ranked according to SUCRA values as follows: NAVA (94%) > SIMV (54%) > PSV (52%) > ASV (40%) > SmartCare/PS (36%) > PAV (24%) (Figure 6).

### 3.6. Publication bias

The correction-comparison funnel plot was created to evaluate the publication bias of duration of mechanical ventilation, duration of ICU stay, hospital stay, ICU mortality, and withdrawal of the ventilator. The result showed that the plot was not symmetric, indicating that there might be publication bias. Results are presented in Supplementary Table S4.

## 4. Discussion

To our knowledge, this is the first network meta-analysis comparing different types of ventilation modes. Mechanical ventilation is a life-support system used to ensure blood gas exchange and to assist the respiratory muscles in ventilating the lungs during the acute phase of lung disease or after surgery. Positive pressure mechanical ventilation is very different from normal physiological breathing (12). This can lead to several negative physiological consequences for the lungs and peripheral organs. Firstly, hemodynamic changes affect cardiovascular function, cerebral perfusion pressure (CPP) and renal venous drainage. Second, the negative effects of mechanical ventilation (compressive stress) on alveolar-capillary membranes and extracellular matrix may cause local and systemic inflammation and promote lung and peripheral organ damage. Third, thoracic hypertension may further impair lung and peripheral organs functioning during controlled and assisted ventilation (14, 35).

We found that there was no significant difference between PSV and other mechanical ventilation modes (NAVA, SIMV, AVS, PAV, Smartcare/PS, and PSV_ATC) in the duration of mechanical ventilation, duration of ICU stay, or hospital stay. SIMV mode could assist the patients in spontaneous breathing under the preset respiratory parameters every minute. ASV is a safe and effective mode based on an adaptive ventilation technique and intelligent synchronization technique, which can be applied in the whole treatment process from intubation to withdrawal of the ventilator (38, 39). Smartcare/PS mode is a new computer-controlled pressure-supported closed-loop ventilation mode, which is commonly used in the stage of withdrawal of the ventilator (40). These conclusions were consistent with a previous meta-analysis (41), which conducted a combined analysis of 20 studies and revealed that there was no significant difference in the duration of mechanical ventilation between NAVA, Smart care/PS, and ASV. In clinical practice, the difficulty in withdrawal of the ventilator could prolong the mechanical ventilation time and increase the risk of mechanical ventilation-related complications, thus prolonging the patients’ stay in the ICU (42). Studies have found that diaphragmatic dysfunction might result in prolonged hospitalization in ICU, failure in withdrawal of the ventilator and increased mortality, while long-term mechanical ventilation could in turn aggravate diaphragmatic dysfunction. These two factors promote and influence each other, which leads to the prolonged hospitalization of patients. In this study, we also found that, compared with PSV, PAV significantly improved the success rate of withdrawal of the ventilator [OR = 3.07, 95% CI (1.21, 8.52)]. PAV mode effectively reduced respiratory work, relieved respiratory muscle fatigue, and improved man–machine coordination and the quality of mechanical ventilation (43, 44). Baudin et al. (45) also proposed that increased respiratory load resulting from man–machine coordination was the main reason for patients’ dependence on the ventilator and failure in withdrawal of the ventilator. Our study also found that the success rate of withdrawal of the ventilator in the PAV group was higher than that in the PSV group, indicating that the PAV mode could reduce respiratory work, relieve respiratory muscle fatigue, improve man–machine coordination and the quality of mechanical ventilation, and consequently assist the patients in the withdrawal of the ventilator. Compared with PSV and PAV, NAVA could reduce ICU mortality (0.63, 95%CI (0.43, 0.93); 0.45, 95%CI (0.21, 0.97)). NAVA is a new assistive ventilation mode directly coupling mechanical ventilation and nerve impulses, which can control the switch of inspiratory and expiratory functions of the ventilator via patients’ own feedback mechanism, thereby reducing the dependence on the ventilator of the ICU patients (46, 47). Some study (48) demonstrated that NAVA could reduce respiratory work and prevent respiratory muscle weakness caused by traditional mechanical ventilation. This new mechanical ventilation mode has significant advantages as follows: (1) since the nerve impulses are monitored, NAVA can effectively shorten the trigger delay, significantly improve human-computer synchronization and avoid false triggering. (2) The breathing mode of NAVA is the most similar to the physiological status. It also adopted the most intelligent tidal volume to maximize man–machine coordination. Firestone et al. (49) also confirmed that respiratory regulation required changes at any time due to the changing condition in patients with respiratory distress syndrome. NAVA mode uses the brain as the most intelligent regulation center to effectively regulate various physiological needs such as respiration, we also need to pay attention to the effects of prone ventilation on the heart, such as reducing heart rate, reducing cardiac load, and increasing cardiac output.

The limitations must be considered when interpreting the conclusions above. First, most studies did not explicitly describe allocation concealment, which might result in selection, implementation, and measurement biases. Second, there was publication bias, which required careful interpretation of the results of this study. Finally, due to the nature of the studied interventions, the blinding of the outcome evaluators was not clearly reported in most enrolled RCTs, thus introducing bias into our analysis. Although in some studies the application of PSV relied on routine care or local guidelines and most assessments were well defined, the risk of deviations in using PSV cannot be completely reduced.

## Conclusion

NAVA can reduce mortality in ICU, and PAV may increase the risk of withdrawal of the ventilator. There was no significant difference between PSV and other mechanical ventilation modes (NAVA, SIMV, AVS, PAV, Smartcare/PS, and PSV_ATC) in the duration of mechanical ventilation, duration of ICU stay, or hospital stay. Due to the limitations, more high-quality studies are needed to verify these findings.

## Data availability statement

The original contributions presented in the study are included in the article/Supplementary material, further inquiries can be directed to the corresponding author.

## Author contributions

MW, XZ, YJ, and YY: concept and design. MW, YG, WZ, HH, and YY: acquisition of data. MW, XZ, YJ, YG, WZ, and HH: statistical analysis. MW, XZ, and YY: interpretation of data and writing original draft. MW, XZ, YJ, YG, WZ, HH, and YY: writing review and editing. All authors contributed to the article and approved the submitted version.

## Conflict of interest

The authors declare that the research was conducted in the absence of any commercial or financial relationships that could be construed as a potential conflict of interest.

## Publisher’s note

All claims expressed in this article are solely those of the authors and do not necessarily represent those of their affiliated organizations, or those of the publisher, the editors and the reviewers. Any product that may be evaluated in this article, or claim that may be made by its manufacturer, is not guaranteed or endorsed by the publisher.
