# Supplementary material for: Comparison of clinical outcomes in critical patients undergoing different mechanical ventilation modes: a systematic review and network meta-analysis
Source: Front Med (Lausanne). 2023 Aug 22;10:1159567. doi: 10.3389/fmed.2023.1159567 (PMC10477667; doi:10.3389/fmed.2023.1159567)
Supplement: Supplementary file 1 [file Table_1.DOCX]

**Supplementary Materials 1:** Search Strategy

| PubMed | |
| --- | --- |
| Search number | Query |
| 11 | (((("Critical Illness"[Mesh]) OR ((((((((((Critical Illness[Title/Abstract]) OR (Critical Illnesses[Title/Abstract])) OR (Illness, Critical[Title/Abstract])) OR (Illnesses, Critical[Title/Abstract])) OR (Critically Ill[Title/Abstract])) OR (Intensive Care Units[Title/Abstract])) OR (Intensive Care Unit[Title/Abstract])) OR (Unit, Intensive Care[Title/Abstract])) OR (ICU Intensive Care Units[Title/Abstract])) OR (ICU[Title/Abstract]))) OR ("Intensive Care Units"[Mesh])) AND ((("Interactive Ventilatory Support"[Mesh]) OR ("High-Frequency Ventilation"[Mesh])) OR (((((((((((((((((((((((((((((((((Interactive Ventilatory Support[Title/Abstract]) OR (Support, Interactive Ventilatory[Title/Abstract])) OR (Ventilatory Support, Interactive[Title/Abstract])) OR (Neurally Adjusted Ventilatory Assist[Title/Abstract])) OR (Proportional Assist Ventilation[Title/Abstract])) OR (Assist Ventilation, Proportional[Title/Abstract])) OR (Ventilation, Proportional Assist[Title/Abstract])) OR (NAVA[Title/Abstract])) OR (PAV[Title/Abstract])) OR (adaptive support ventilation[Title/Abstract])) OR (ASV[Title/Abstract])) OR (intellivent-ASV[Title/Abstract])) OR (High-Frequency Ventilation[Title/Abstract])) OR (High-Frequency Ventilations[Title/Abstract])) OR (Ventilations, High-Frequency[Title/Abstract])) OR (Ventilation, High Frequency[Title/Abstract])) OR (Ventilation, High-Frequency[Title/Abstract])) OR (High Frequency Ventilation[Title/Abstract])) OR (High Frequency Ventilations[Title/Abstract])) OR (Ventilations, High Frequency[Title/Abstract])) OR (High-Frequency Oscillation Ventilation[Title/Abstract])) OR (High-Frequency Oscillation Ventilations[Title/Abstract])) OR (Oscillation Ventilation, High-Frequency[Title/Abstract])) OR (Oscillation Ventilations, High-Frequency[Title/Abstract])) OR (Ventilation, High-Frequency Oscillation[Title/Abstract])) OR (Ventilations, High-Frequency Oscillation[Title/Abstract])) OR (High Frequency Oscillation Ventilation[Title/Abstract])) OR (High-Frequency Positive Pressure Ventilation[Title/Abstract])) OR (High Frequency Positive Pressure Ventilation[Title/Abstract])) OR (Smartcare/PS[Title/Abstract])) OR (PSV[Title/Abstract])) OR (Synchronized Intermittent Mandatory Ventilation[Title/Abstract])) OR (SIMV[Title/Abstract])))) AND (randomized controlled trial[Publication Type] OR randomized[Title/Abstract] OR placebo[Title/Abstract]) |
| 10 | randomized controlled trial[Publication Type] OR randomized[Title/Abstract] OR placebo[Title/Abstract] |
| 9 | ((("Critical Illness"[Mesh]) OR ((((((((((Critical Illness[Title/Abstract]) OR (Critical Illnesses[Title/Abstract])) OR (Illness, Critical[Title/Abstract])) OR (Illnesses, Critical[Title/Abstract])) OR (Critically Ill[Title/Abstract])) OR (Intensive Care Units[Title/Abstract])) OR (Intensive Care Unit[Title/Abstract])) OR (Unit, Intensive Care[Title/Abstract])) OR (ICU Intensive Care Units[Title/Abstract])) OR (ICU[Title/Abstract]))) OR ("Intensive Care Units"[Mesh])) AND ((("Interactive Ventilatory Support"[Mesh]) OR ("High-Frequency Ventilation"[Mesh])) OR (((((((((((((((((((((((((((((((((Interactive Ventilatory Support[Title/Abstract]) OR (Support, Interactive Ventilatory[Title/Abstract])) OR (Ventilatory Support, Interactive[Title/Abstract])) OR (Neurally Adjusted Ventilatory Assist[Title/Abstract])) OR (Proportional Assist Ventilation[Title/Abstract])) OR (Assist Ventilation, Proportional[Title/Abstract])) OR (Ventilation, Proportional Assist[Title/Abstract])) OR (NAVA[Title/Abstract])) OR (PAV[Title/Abstract])) OR (adaptive support ventilation[Title/Abstract])) OR (ASV[Title/Abstract])) OR (intellivent-ASV[Title/Abstract])) OR (High-Frequency Ventilation[Title/Abstract])) OR (High-Frequency Ventilations[Title/Abstract])) OR (Ventilations, High-Frequency[Title/Abstract])) OR (Ventilation, High Frequency[Title/Abstract])) OR (Ventilation, High-Frequency[Title/Abstract])) OR (High Frequency Ventilation[Title/Abstract])) OR (High Frequency Ventilations[Title/Abstract])) OR (Ventilations, High Frequency[Title/Abstract])) OR (High-Frequency Oscillation Ventilation[Title/Abstract])) OR (High-Frequency Oscillation Ventilations[Title/Abstract])) OR (Oscillation Ventilation, High-Frequency[Title/Abstract])) OR (Oscillation Ventilations, High-Frequency[Title/Abstract])) OR (Ventilation, High-Frequency Oscillation[Title/Abstract])) OR (Ventilations, High-Frequency Oscillation[Title/Abstract])) OR (High Frequency Oscillation Ventilation[Title/Abstract])) OR (High-Frequency Positive Pressure Ventilation[Title/Abstract])) OR (High Frequency Positive Pressure Ventilation[Title/Abstract])) OR (Smartcare/PS[Title/Abstract])) OR (PSV[Title/Abstract])) OR (Synchronized Intermittent Mandatory Ventilation[Title/Abstract])) OR (SIMV[Title/Abstract]))) |
| 8 | (("Critical Illness"[Mesh]) OR ((((((((((Critical Illness[Title/Abstract]) OR (Critical Illnesses[Title/Abstract])) OR (Illness, Critical[Title/Abstract])) OR (Illnesses, Critical[Title/Abstract])) OR (Critically Ill[Title/Abstract])) OR (Intensive Care Units[Title/Abstract])) OR (Intensive Care Unit[Title/Abstract])) OR (Unit, Intensive Care[Title/Abstract])) OR (ICU Intensive Care Units[Title/Abstract])) OR (ICU[Title/Abstract]))) OR ("Intensive Care Units"[Mesh]) |
| 7 | (((((((((Critical Illness[Title/Abstract]) OR (Critical Illnesses[Title/Abstract])) OR (Illness, Critical[Title/Abstract])) OR (Illnesses, Critical[Title/Abstract])) OR (Critically Ill[Title/Abstract])) OR (Intensive Care Units[Title/Abstract])) OR (Intensive Care Unit[Title/Abstract])) OR (Unit, Intensive Care[Title/Abstract])) OR (ICU Intensive Care Units[Title/Abstract])) OR (ICU[Title/Abstract]) |
| 6 | "Intensive Care Units"[Mesh] |
| 5 | "Critical Illness"[Mesh] |
| 4 | (("Interactive Ventilatory Support"[Mesh]) OR ("High-Frequency Ventilation"[Mesh])) OR (((((((((((((((((((((((((((((((((Interactive Ventilatory Support[Title/Abstract]) OR (Support, Interactive Ventilatory[Title/Abstract])) OR (Ventilatory Support, Interactive[Title/Abstract])) OR (Neurally Adjusted Ventilatory Assist[Title/Abstract])) OR (Proportional Assist Ventilation[Title/Abstract])) OR (Assist Ventilation, Proportional[Title/Abstract])) OR (Ventilation, Proportional Assist[Title/Abstract])) OR (NAVA[Title/Abstract])) OR (PAV[Title/Abstract])) OR (adaptive support ventilation[Title/Abstract])) OR (ASV[Title/Abstract])) OR (intellivent-ASV[Title/Abstract])) OR (High-Frequency Ventilation[Title/Abstract])) OR (High-Frequency Ventilations[Title/Abstract])) OR (Ventilations, High-Frequency[Title/Abstract])) OR (Ventilation, High Frequency[Title/Abstract])) OR (Ventilation, High-Frequency[Title/Abstract])) OR (High Frequency Ventilation[Title/Abstract])) OR (High Frequency Ventilations[Title/Abstract])) OR (Ventilations, High Frequency[Title/Abstract])) OR (High-Frequency Oscillation Ventilation[Title/Abstract])) OR (High-Frequency Oscillation Ventilations[Title/Abstract])) OR (Oscillation Ventilation, High-Frequency[Title/Abstract])) OR (Oscillation Ventilations, High-Frequency[Title/Abstract])) OR (Ventilation, High-Frequency Oscillation[Title/Abstract])) OR (Ventilations, High-Frequency Oscillation[Title/Abstract])) OR (High Frequency Oscillation Ventilation[Title/Abstract])) OR (High-Frequency Positive Pressure Ventilation[Title/Abstract])) OR (High Frequency Positive Pressure Ventilation[Title/Abstract])) OR (Smartcare/PS[Title/Abstract])) OR (PSV[Title/Abstract])) OR (Synchronized Intermittent Mandatory Ventilation[Title/Abstract])) OR (SIMV[Title/Abstract])) |
| 3 | ((((((((((((((((((((((((((((((((Interactive Ventilatory Support[Title/Abstract]) OR (Support, Interactive Ventilatory[Title/Abstract])) OR (Ventilatory Support, Interactive[Title/Abstract])) OR (Neurally Adjusted Ventilatory Assist[Title/Abstract])) OR (Proportional Assist Ventilation[Title/Abstract])) OR (Assist Ventilation, Proportional[Title/Abstract])) OR (Ventilation, Proportional Assist[Title/Abstract])) OR (NAVA[Title/Abstract])) OR (PAV[Title/Abstract])) OR (adaptive support ventilation[Title/Abstract])) OR (ASV[Title/Abstract])) OR (intellivent-ASV[Title/Abstract])) OR (High-Frequency Ventilation[Title/Abstract])) OR (High-Frequency Ventilations[Title/Abstract])) OR (Ventilations, High-Frequency[Title/Abstract])) OR (Ventilation, High Frequency[Title/Abstract])) OR (Ventilation, High-Frequency[Title/Abstract])) OR (High Frequency Ventilation[Title/Abstract])) OR (High Frequency Ventilations[Title/Abstract])) OR (Ventilations, High Frequency[Title/Abstract])) OR (High-Frequency Oscillation Ventilation[Title/Abstract])) OR (High-Frequency Oscillation Ventilations[Title/Abstract])) OR (Oscillation Ventilation, High-Frequency[Title/Abstract])) OR (Oscillation Ventilations, High-Frequency[Title/Abstract])) OR (Ventilation, High-Frequency Oscillation[Title/Abstract])) OR (Ventilations, High-Frequency Oscillation[Title/Abstract])) OR (High Frequency Oscillation Ventilation[Title/Abstract])) OR (High-Frequency Positive Pressure Ventilation[Title/Abstract])) OR (High Frequency Positive Pressure Ventilation[Title/Abstract])) OR (Smartcare/PS[Title/Abstract])) OR (PSV[Title/Abstract])) OR (Synchronized Intermittent Mandatory Ventilation[Title/Abstract])) OR (SIMV[Title/Abstract]) |
| 2 | "High-Frequency Ventilation"[Mesh] |
| 1 | "Interactive Ventilatory Support"[Mesh] |
