# Supplementary material for: Comparison of clinical outcomes in critical patients undergoing different mechanical ventilation modes: a systematic review and network meta-analysis
Source: Front Med (Lausanne). 2023 Aug 22;10:1159567. doi: 10.3389/fmed.2023.1159567 (PMC10477667; doi:10.3389/fmed.2023.1159567)
Supplement: Supplementary file 2 [file Table_2.DOCX]

| MD (95%CI) | | | | | | | |
| --- | --- | --- | --- | --- | --- | --- | --- |
| ASV | ASV |  |  |  |  |  |  |
| NAVA | 0 (0, 157442.69) | NAVA |  |  |  |  |  |
| PAV | 0 (0, 136707.91) | 0.02 (0, 5481971049.59) | PAV |  |  |  |  |
| PSV | 0 (0, 0.02) | 0 (0, 11410.21) | 0.01 (0, 172759242.51) | PSV |  |  |  |
| PSV_ATC | 0 (0, 399386.4) | 0 (0, 775081115886.56) | 0 (0, 569656917904357) | 0 (0, 74559687134994.7) | PSV_ATC |  |  |
| SIMV | 1.06 (0, 2817288930964.92) | 483353.64 (0, 6.77802758918704e+21) | 28405290.48 (0, 8.70013569652499e+24) | 2986754065.09 (0, 3.09555321932525e+23) | 2379023354133.91 (0, 4.18138663478942e+34) | SIMV |  |
| SmartCare_PS | 0 (0, 5854754.2) | 0.87 (0, 6204340302184.63) | 52.03 (0, 12960218536309962) | 5206.81 (0, 64909530954156.2) | 4474943.6 (0, 3.47605695426381e+26) | 0 (0, 7555479454.21) | SmartCare_PS |

F1: Duration of mechanical ventilation

F2: Duration of ICU stay

| MD (95%CI) | | | | | | | |
| --- | --- | --- | --- | --- | --- | --- | --- |
| ASV | ASV |  |  |  |  |  |  |
| NAVA | 2.31 (0, 70963.03) | NAVA |  |  |  |  |  |
| PAV | 0.18 (0, 3789.89) | 0.08 (0, 716.53) | PAV |  |  |  |  |
| PSV | 0.15 (0, 324.89) | 0.06 (0, 69.99) | 0.83 (0, 576.24) | PSV |  |  |  |
| PSV_ATC | 0.24 (0, 362678.53) | 0.1 (0, 127486.43) | 1.33 (0, 1271488.78) | 1.64 (0, 265789.44) | PSV_ATC |  |  |
| SIMV | 0.07 (0, 848.61) | 0.03 (0, 3745.14) | 0.38 (0, 35046.72) | 0.46 (0, 5206.66) | 0.28 (0, 1231414.06) | SIMV |  |
| SmartCare_PS | 2.92 (0, 55238507.3) | 1.3 (0, 20186703.93) | 17.26 (0, 188041779.35) | 20.39 (0, 41401227.03) | 12.06 (0, 3905105731.92) | 43.65 (0, 2604801389.77) | SmartCare_PS |

F3: Hospital stay

| MD (95%CI) | | | | | | | |
| --- | --- | --- | --- | --- | --- | --- | --- |
| ASV | ASV |  |  |  |  |  |  |
| NAVA | 2.29 (0, 62932.37) | NAVA |  |  |  |  |  |
| PAV | 0.18 (0, 3744.98) | 0.08 (0, 676.47) | PAV |  |  |  |  |
| PSV | 0.15 (0, 312.13) | 0.07 (0, 65.59) | 0.83 (0, 529.35) | PSV |  |  |  |
| PSV_ATC | 0.25 (0, 344391.96) | 0.11 (0, 102434.98) | 1.36 (0, 1025966.24) | 1.65 (0, 240339.75) | PSV_ATC |  |  |
| SIMV | 0.07 (0, 741.53) | 0.03 (0, 2878.6) | 0.37 (0, 28696.7) | 0.46 (0, 4405.2) | 0.27 (0, 945723.83) | SIMV |  |
| SmartCare_PS | 2.91 (0, 49352346.53) | 1.27 (0, 15935596) | 16.16 (0, 146918454.03) | 19.51 (0, 35099443.04) | 11.32 (0, 3264369093.24) | 42.48 (0, 2269197913.41) | SmartCare_PS |

F5: Successfully weaned

| OR (95%CI) | | | | |
| --- | --- | --- | --- | --- |
| ASV | ASV |  |  |  |
| NAVA | 0.79 (0.16, 4.16) | NAVA |  |  |
| PAV | 0.43 (0.07, 2.7) | 0.55 (0.17, 1.65) | PAV |  |
| PSV | 1.34 (0.29, 6.44) | 1.7 (0.97, 3) | 3.07 (1.21, 8.52) | PSV |

F6: ICU mortality

| OR (95%CI) | | | | | | |
| --- | --- | --- | --- | --- | --- | --- |
| ASV | ASV |  |  |  |  |  |
| NAVA | 1.83 (0.77, 4.37) | NAVA |  |  |  |  |
| PAV | 0.83 (0.29, 2.34) | 0.45 (0.21, 0.97) | PAV |  |  |  |
| PSV | 1.15 (0.53, 2.53) | 0.63 (0.43, 0.93) | 1.39 (0.7, 2.82) | PSV |  |  |
| SIMV | 1.17 (0.48, 2.89) | 0.64 (0.35, 1.16) | 1.42 (0.62, 3.27) | 1.01 (0.65, 1.59) | SIMV |  |
| SmartCare_PS | 0.92 (0.25, 3.34) | 0.5 (0.17, 1.52) | 1.11 (0.32, 3.88) | 0.8 (0.28, 2.24) | 0.79 (0.25, 2.43) | SmartCare_PS |
