# Supplementary material for: Comparison of clinical outcomes in critical patients undergoing different mechanical ventilation modes: a systematic review and network meta-analysis
Source: Front Med (Lausanne). 2023 Aug 22;10:1159567. doi: 10.3389/fmed.2023.1159567 (PMC10477667; doi:10.3389/fmed.2023.1159567)
Supplement: Supplementary file 3 [file Table_3.DOCX]

The ranking diagram of duration of mechanical ventilation





The ranking diagram of duration of ICU stay





The ranking diagram of hospital stay
