# Supplementary material for: Comparison of clinical outcomes in critical patients undergoing different mechanical ventilation modes: a systematic review and network meta-analysis
Source: Front Med (Lausanne). 2023 Aug 22;10:1159567. doi: 10.3389/fmed.2023.1159567 (PMC10477667; doi:10.3389/fmed.2023.1159567)
Supplement: Supplementary file 4 [file Table_4.DOCX]

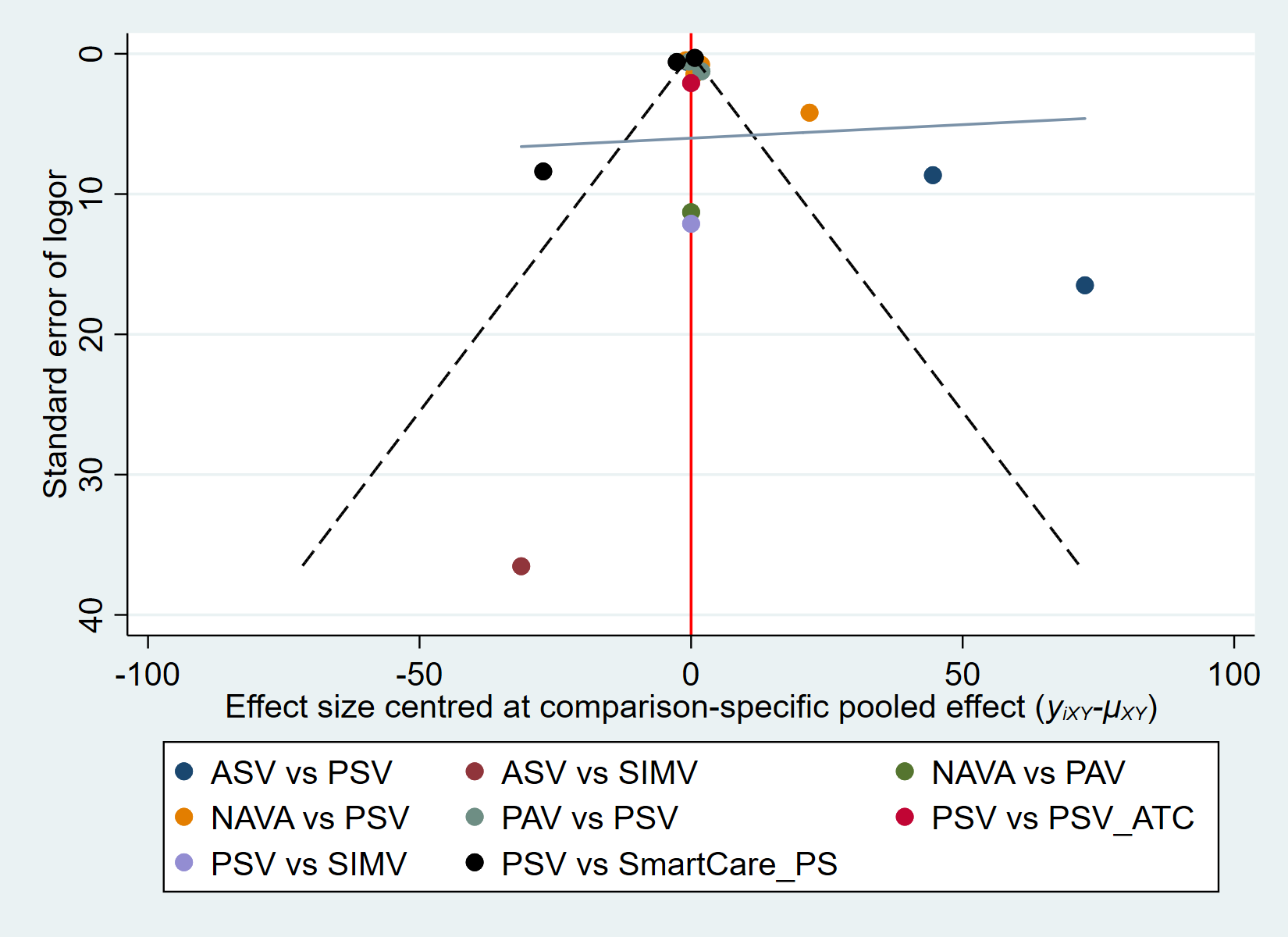


**Duration of mechanical ventilation funnel plot**


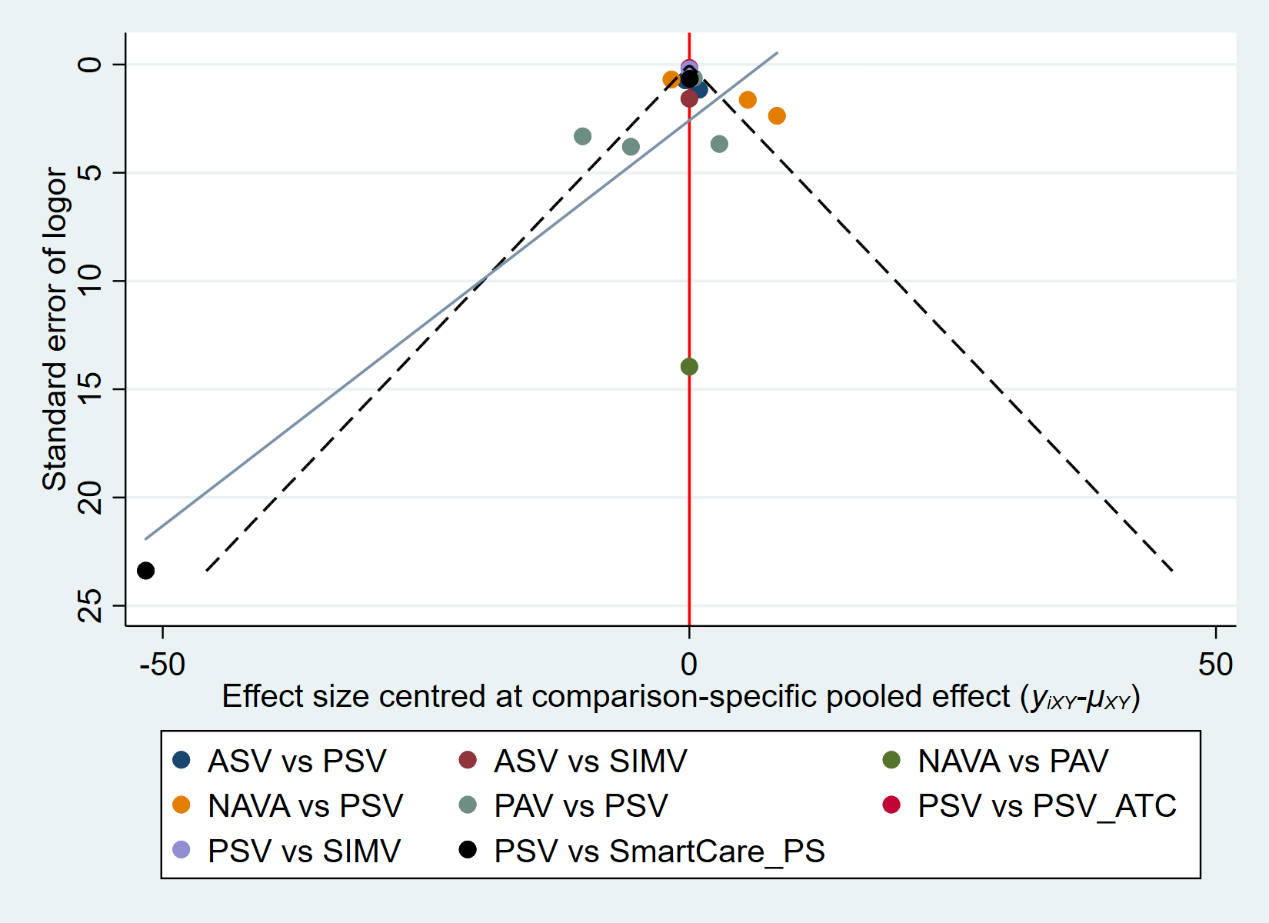


**Duration of ICU stay funnel plot**


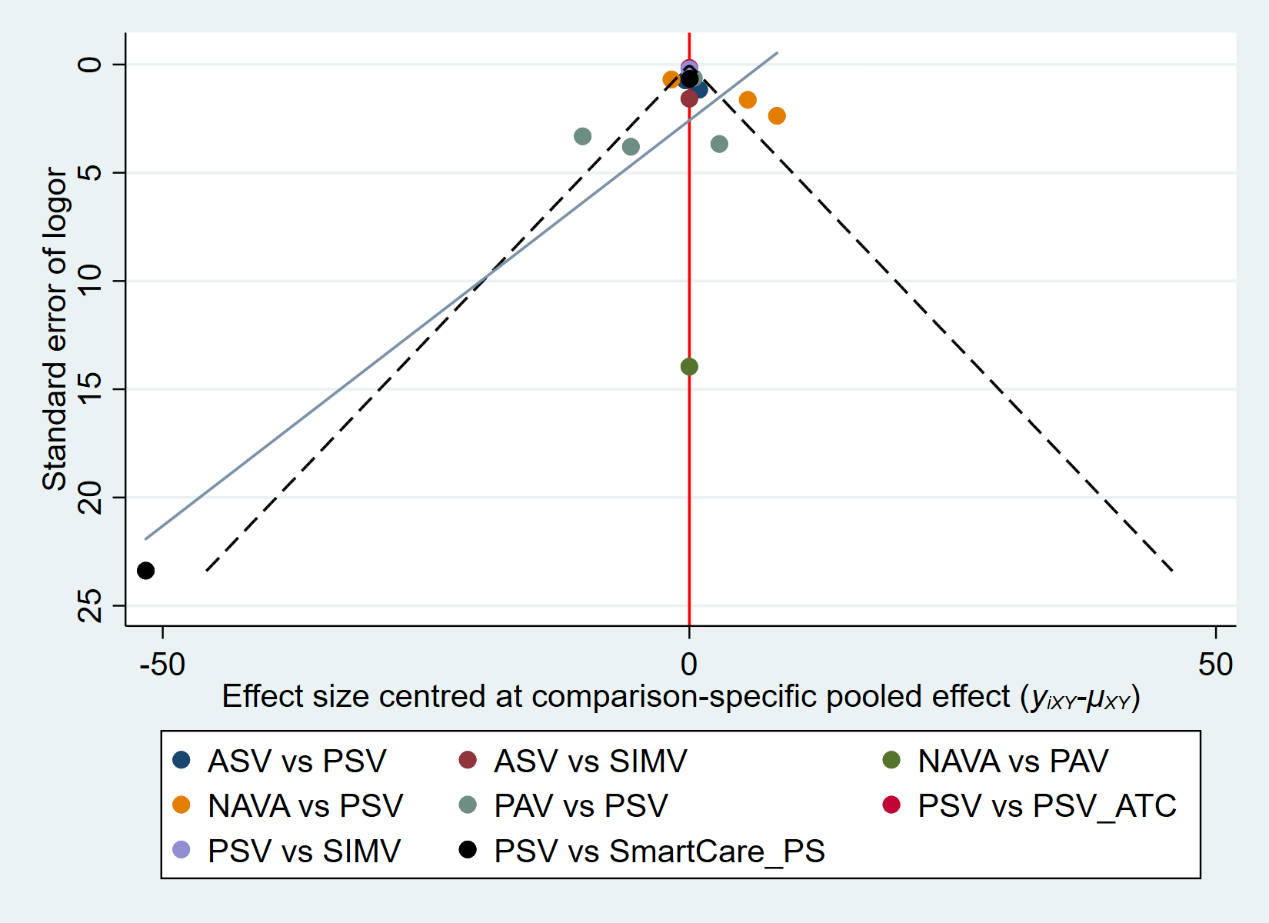


**Hospital stay funnel plot**


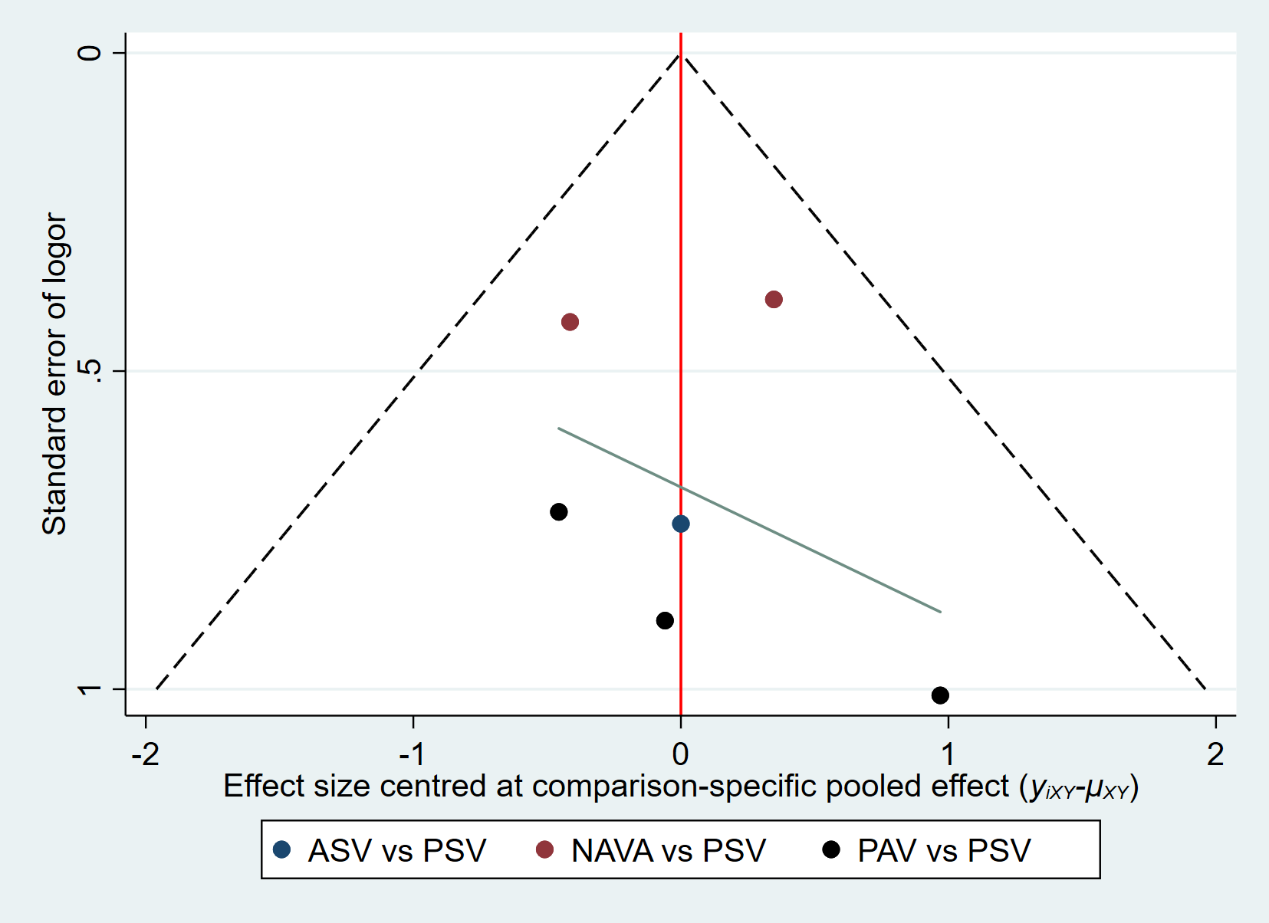


**Successfully weaned from the ventilator funnel plot**


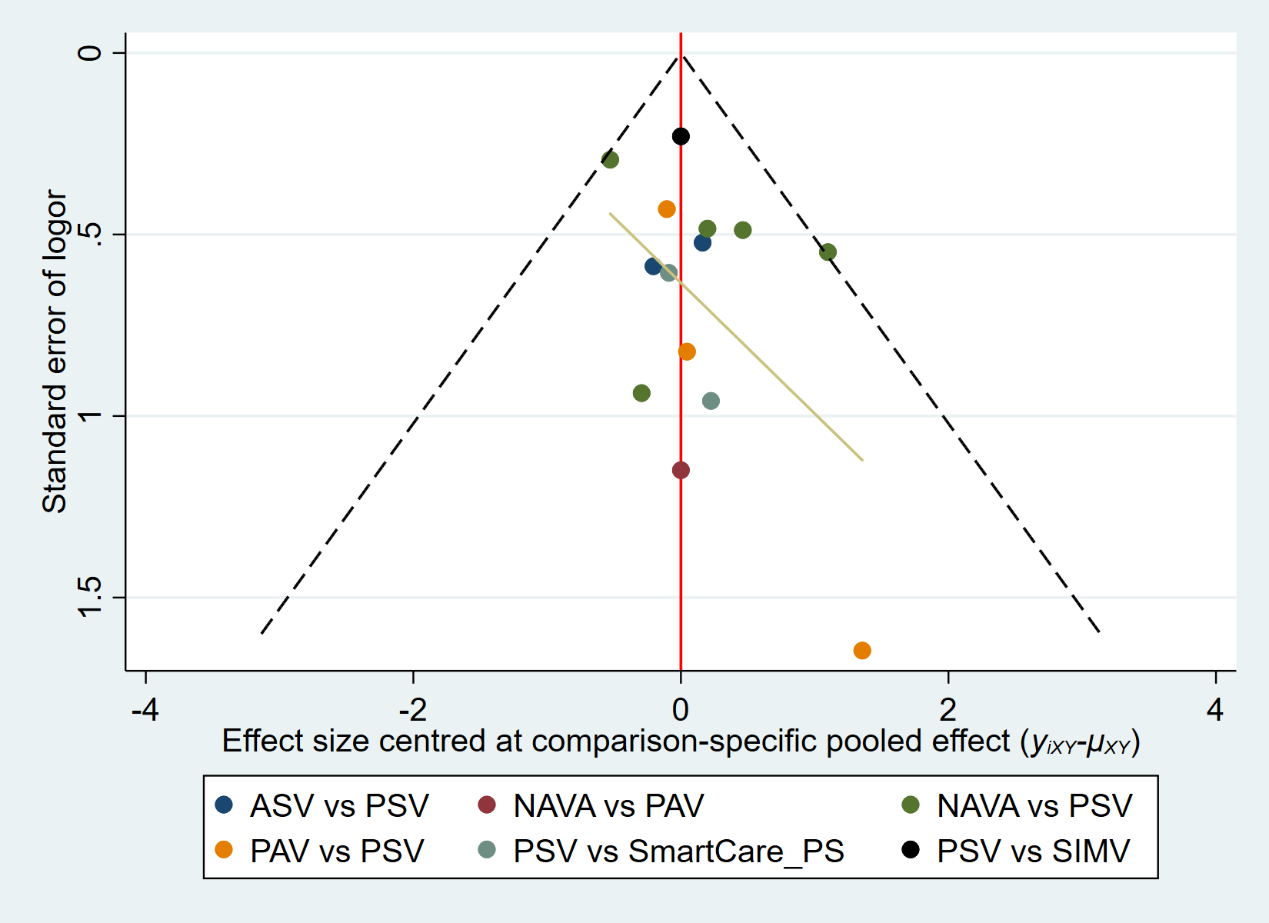


**ICU mortality funnel plot**
